# Eulachon Oil

**Published:** 1885-01

**Authors:** 


### Eulachon Oil.

Dr. E. L. Shurley, of Detroit (*New York Medical Journal*, Nov. 29, 1884), furnishes interesting information concerning eulachon or "candle-fish" oil. He has found it an efficient agent in the treatment of phthisis and other wasting diseases, and thinks it bids fair to become a rival of cod-liver oil in these cases. He has used it in twelve cases of phthisis,—seven in hospital, and five in private practice,—and, from the summary of these cases, which he publishes, it would appear that eulachon oil is, at least, not inferior to cod-liver oil in the class of cases to which it is adapted, and where patients can tolerate the ingestion of an oil.

In most of Dr. Shurley's cases, cod-liver oil had been previously administered, so that he was enabled to compare the relative merits of the two oils in the same individuals. The points of favor which he seems to fairly claim for the new fish oil are, first, its superior digestibility over that of cod-liver oil; second, its lessened liability to produce diarrhœa; and, third, its less disagreeable properties. The price, a matter of considerable moment to most patients who require such remedies, is reported to be as low if not lower than cod-liver oil.

For ourselves, we confess to the fact that we are, year by year, growing into the habit of recommending cod-liver oil less frequently, not because we do not value it highly as a food, *per se*, but because of the many difficulties surrounding its successful administration. The patients who need it most seem to be the least able to ingest, much less digest, it in sufficient quantities to render it beneficial in arresting or restoring tissue-waste. The various emulsions of commerce are of doubtful virtue to say the least; it were better to administer either the plain oil of a sweet and pure variety, or to extemporize an emulsion with yolk of egg, glycerine, and any agreeable flavoring combination.

Our readers are referred to an article by Dr. A. B. Lyons in the *Therapeutic Gazette*, September, 1884, for a description of the physical and chemical properties of eulachon oil. This oil is said to contain a substance analogous to paraffine, which may account for its apparent superiority to cod-liver oil, as claimed by Dr. Shurley. From his endorsement of this new aliment we are inclined to regard it as a medicinal agent of efficiency in phthisis and similar wasting diseases, and hope it may be put upon trial in our public institutions with a view to thoroughly and fairly test its merits and fix its status.

